# Biological variation of baseline serum cortisol concentration in healthy dogs, part 1: variance components

**DOI:** 10.3389/fvets.2026.1805880

**Published:** 2026-03-31

**Authors:** Sam Wicker, Sydney Craig, Julia Albright, Cary Springer, Kathleen Freeman, Luca Giori

**Affiliations:** 1 College of Veterinary Medicine, Biomedical and Diagnostic Sciences, University of Tennessee, Knoxville, TN, United States; 2 College of Veterinary Medicine, Small Animal Clinical Sciences, University of Tennessee, Knoxville, TN, United States; 3 Office of Innovative Technologies, Research Computing Support, University of Tennessee, Knoxville, TN, United States; 4 Veterinary Information Network, Davis, CA, United States

**Keywords:** baseline cortisol, biological variation, canine (dog), endocrinology, healthy dogs

## Abstract

**Introduction:**

The measurement of baseline cortisol concentrations in dogs is affected by within-subject (individual, CV_I_) and between-subject (group, CV_G_) variability, together called “biological variation,” as well as analytical variability (CV_A_).

**Methods:**

Baseline cortisol concentrations analyzed in duplicate from 18 healthy, acclimated, client-owned dogs over a 6-week period were used to quantify biological variation for this measurand.

**Results:**

Individual variation and group variation were CV_I_ = 32.41% and CV_G_ = 37.70%, and analytical variation (CV_A_) was 6.86%. Cortisol concentrations were not associated with age, breed, sex, neuter status, or venipuncture site (jugular vs. cephalic). Baseline cortisol concentrations were not associated with date of sampling as the study progressed.

**Discussion:**

Biological variation of baseline cortisol in dogs can be used to help interpret individual cortisol results and when establishing laboratory quality goals. Veterinarians should be aware of the potential biological variation of baseline cortisol measurements in dogs.

## Introduction

Cortisol is a key regulator of physiological homeostasis and an important biomarker in the diagnosis and management of some endocrine disorders. Measuring baseline cortisol concentration from a single serum sample is straightforward, minimally stressful, and may identify changes to an individual’s homeostatic setpoint (HSP) for cortisol due to disease. This concentration can be used as an initial evaluation to identify potential endocrine abnormalities and guide the need for more comprehensive testing (1–4).

Variability in baseline cortisol concentrations around an HSP can occur between dogs based on age, sex, breed, and environmental factors. For example, Gal et al. (5) found differences in mean serum cortisol concentrations after ACTH stimulation (tetracosactrin injection) between male and female dogs and several studies have found differences in cortisol levels based on breed or body weight (6–8). In humans, genetics and environmental factors such as sleep consistency also contribute to baseline cortisol concentrations (9, 10). Given the variety of factors that may affect an individual dog’s HSP for cortisol, a result within normal limits for one individual might reflect a pathophysiological change for another.

Natural fluctuations around the HSP also occur within individual dogs, as cortisol concentrations exhibit significant circadian rhythms and pulsatile secretion patterns. In people, cortisol levels peak in the morning with smaller secondary peaks seen after meals (11, 12). In contrast to humans, serum and saliva cortisol concentrations peak in the early afternoon in dogs (13). Variation between subjects (or group variation, CV_G_) and variation within a subject (or individual variation, CV_I_) are often referred to together as “biological variation.”

Analytical methods add a third layer of variability. This variation is inherent to all laboratory test methods and is often evident when comparing results between individual analyzers. Pre-analytical factors might also affect measured baseline cortisol levels (39). Venipuncture-induced stress is known to influence various blood measurands (14, 15) and postural changes or the site of blood draw may introduce pre-analytical variability in laboratory measurements (16).

Awareness of biological variation, analytical variation, and their effects on cortisol concentrations can assist interpretation of individual results and serial subject data, avoid overinterpretation of a single test result, reveal limitations of a clinical test, and guide laboratories in establishing quality goals (17). Studies quantifying biological variation involve serial sample collections from a representative group of subjects and provide information about individual variability (CV_I_), group variability (CV_G_), and analytical variability (CV_A_). Guidelines for conducting and reporting biological variation studies were published in 2017 to standardize and enhance the quality of published information (18). These guidelines facilitate the review and selection of publications as standard references, ensuring consistency and reliability in data reporting.

The primary objective of this study was to evaluate the biological variation of weekly baseline cortisol concentrations in healthy dogs over a 6-week period. The effect of the sampling site (jugular vs. cephalic) and sequential sampling (first or second sample) on measured cortisol concentrations was also explored. To the authors’ knowledge, this is the first study to investigate the biological variation of baseline cortisol in healthy dogs.

## Methods

Healthy staff- and student-owned dogs from the University of Tennessee College of Veterinary Medicine were recruited for this study. Participation in the study was voluntary, and owners were asked to sign an informed consent form during the first week of the study. The study design and protocol were approved by the Institutional Animal Care and Use Committee before commencement (protocol #3042). To be included, dogs had to be in good health, amenable to venipuncture, weigh over 10 kg, and be between 1 and 10 years of age. Health status was owner-reported and confirmed with complete physical exam as well as complete blood cell count and serum biochemistry performed the first week of the study. Exclusion criteria were illness or administration of any medication, excluding parasite preventatives, 4 weeks prior to the start of the study or during the 6-weeks of sampling.

Sampling was carried out for each participant once a week for 6 weeks in the summer of 2024 (from June 10th to July 24th). Each participant was sampled weekly, on a consistent weekday, and within the same 45-min window for the duration of the study. Appointment times spanned 11:45 a.m.–3:30 p.m. with the earliest sample taken at 12:05 p.m. and the latest sample taken at 3:20 p.m. Eastern Daylight Time.

All sampling occurred within the same room (approximately 6 m × 3 m) with consistent staff members (3 total). The week before the study, each dog came for a 20-min session to acclimate to the examination environment, phlebotomists, and data collector (SC). During these sessions, dogs were given time to investigate the environment and interact with technicians and their owners through petting, food treats, and toy play. For the 6 collection sessions, each participant was given a 30-min weekly appointment. The first 20 min of each appointment were for acclimation, and the last 10 min were reserved for sample collection and processing. During the initial acclimation period of each of the experimental sessions, the dogs were allowed to investigate the room but were encouraged to rest quietly. No food or play occurred before the venipuncture, but food was often provided as a positive distractor during the blood draw. Food and play were encouraged after the venipuncture to reduce negative emotional associations with the procedure.

Owners were allowed to stay or could choose to drop their dog off, provided their choice was consistent each week. There was one makeup week in the fifth overall week of the study where several participants who had an unsuccessful blood draw or owner schedule conflict returned to ensure completion of six samples per participant while the other participants skipped this week of sampling. Owners were asked each week about the health and activity of their animals from the week prior and were encouraged to keep their routine as consistent as possible for the day of each visit.

For the first 5 collection appointments, the dogs were gently restrained, and 2–4 mL of blood were obtained from the jugular vein by one of two experienced veterinary technicians using 20- or 22- gauge needles and 6 mL syringes. During the final week, 2 mL of blood was drawn from both a jugular vein and a medial cephalic vein in a counterbalanced order determined by a random number generator. Blood was collected in non-gel serum tubes and was allowed to clot for 30 min before centrifugation at 3,400 rpm (1,800×*g*) for 12 min. Serum samples were visually inspected for hemolysis and lipemia, placed in a 4C° refrigerator within 1 h of blood collection, and then 0.3 μL were aliquoted into two 1.5 mL cryotubes and placed in a -80C° freezer within 4 h of blood collection. Storage time ranged from 1 to 45 days.

All hormone analyses were performed in 1 day on sequentially thawed serum samples to maintain a similar thawing time between each sample. Cortisol was measured in duplicate using a chemiluminescence immunoassay system (IMMULITE 2000 XPi, Siemens Healthcare Diagnostics Products Ltd., Los Angeles, CA) with a veterinary cortisol kit (VCO; Siemens Healthcare Diagnostics Products Ltd., Los Angeles, CA). Although samples were measured in duplicate, this analyzer performs 12 replicate measurements for each sample, discards the highest and lowest sample, and reports the mean of the 10 remaining measurements. Hormone measurements were performed in accordance with the manufacturer’s instructions by laboratory personnel blinded to dog identity and time points. The manufacturer’s instructions state that hemolysis with hemoglobin concentrations up to 384 mg/dL or lipemia with triglyceride concentrations up to 3,000 mg/dL have no effect on results, within the precision of the assay. Samples may be stored for up to 3 months below −20 °C.

## Statistical analysis

Mixed model analyses of variance repeated measures (ANOVAs) were run to compare week and demographic differences, with week and demographic as fixed factors, participant ID letters as a random factor and a diagonal covariance structure was used. All statistical analyses were analyzed in JMP Pro 17.0.0 (JMP Statistical Discovery, Cary, NC, USA). Significance was set at *p* < 0.05.

The biological variation parameters CV_G_, CV_I_, and CV_A_ were derived using a Restricted Maximum Likelihood (REML) mixed model analysis of variance (ANOVA). In this model, participant ID letters were treated as random effects, with weeks nested within each participant ID to estimate analytical, inter-individual, and intra-individual variances. After analysis, a Shapiro–Wilk test confirmed that the residuals followed a normal (Gaussian) distribution with constant variance.

Specifically, data from all dogs underwent nested ANOVA, yielding between-dog variance (S^2^_inter), within-dog variance (S^2^_intra), and analytical variance (S^2^_a). These variances were then converted into CV_G_, CV_I_, and CV_A_, respectively, using the overall mean value.

Mixed model repeated measures ANOVA were performed to test for differences between the blood collected from the jugular and cephalic veins at week 6, as well as to test for an order effect. Collection site, sequence and period were set as fixed factors, ID nested within sequence as a random factor and a scaled identity covariance structure was used.

## Results

Twenty (20) dogs were initially enrolled in the study, but two dogs were excluded due to excessive resistance to venipuncture. Data from the remaining 18 dogs were used in the statistical analysis. The mean ± standard deviation (±SD) weight of the dogs was 26.89 kg ± 10.09 kg. The mean (±SD) age of the dogs was 5.4 years ± 2.5 years. Participants included male castrated (*n* = 7), male intact (*n* = 1), female castrated (*n* = 9), and female intact (*n* = 1). Breeds included Standard Poodle (*n* = 2), German Shepherd (*n* = 2), Great Pyrenees (*n* = 1), Irish Setter (*n* = 1), Rough Coated Collie (*n* = 1), Corgi (*n* = 1), Golden Retriever (*n* = 2), American Pitbull terrier (*n* = 1), American Bully (*n* = 1) and mixed breed (*n* = 6). (Table 1). Minimal hemolysis was identified visually in four samples and no lipemia was noted, with no samples excluded on this basis. One dog (Dog H) has only 5 results due to an instrument error during analysis of the sample from week 4. Also, blood collection from the cephalic vein of dog C during week six was unsuccessful due to insufficient sample volume. As a result, the paired value was excluded from the statistical comparison of the site of collection.

**Table 1:** Cortisol concentrations (μg/dL) for the 18 healthy dogs enrolled in this study.

Animal ID	Age (years)	Breed	Sex/neuter status	Week 1	Week 2	Week 3	Week 4	Week 5	Week 6-J	Week 6-C
A	6	Mixed	F/S	2.11	2.13	1.63	2.37	2.59	1.85	1.77
B	4	German Shepherd Dog	M/I	0.85	0.87	1.09	1.18	0.83	0.57	0.54
C	8	Mixed	F/S	2.87	1.42	1.34	1.24	1.65	2.86	-
D	8	Mixed	M/N	1.68	1.05	1.32	1.38	1.53	1.15	0.69
E	2	Mixed	M/N	1.54	1.16	1.02	1.08	1.18	1.02	0.73
F	5	Corgi	M/N	1.26	1.71	0.91	1.18	1.10	0.76	1.08
G	4.5	Irish Setter	F/I	0.97	2.01	1.13	1.71	1.14	1.21	1.14
H	6	Mixed	F/S	3.32	3.99	3.40	-	4.43	3.26	3.03
I	3	Standard Poodle	F/S	2.73	1.23	1.46	2.17	2.18	3.44	3.10
J	8	Pitbull Terrier	F/S	2.05	3.94	2.68	3.39	3.90	1.93	1.86
K	5	American Bully	F/S	1.55	1.75	0.89	1.60	1.43	1.31	1.33
L	2	Standard Poodle	M/N	1.95	4.09	2.59	3.20	2.25	2.10	2.34
M	2.5	Great Pyrenees	M/N	3.70	4.04	4.34	4.06	3.20	3.79	3.62
O	5	Rough Coated Collie	M/N	4.21	4.43	0.82	1.42	2.26	2.84	2.72
P	7	Golden Retriever	F/S	1.21	2.38	1.90	1.74	2.84	1.71	1.75
Q	9	Mixed	F/S	2.76	2.07	1.23	1.62	1.93	1.23	1.29
S	10	Golden Retriever	M/N	2.69	2.15	2.82	2.17	2.03	1.71	1.83
T	2	German Shepherd Dog	F/S	2.02	0.41	4.21	3.14	2.34	3.40	3.35

Weekly values represent the means of duplicate measurements. Week 6-J and Week 6-C indicate cortisol levels from samples collected from the jugular and cephalic veins, respectively. For sex/neuter status, abbreviations represent: F, female; M, male, S, spayed; I, intact; N, neutered.

**Figure 1:**
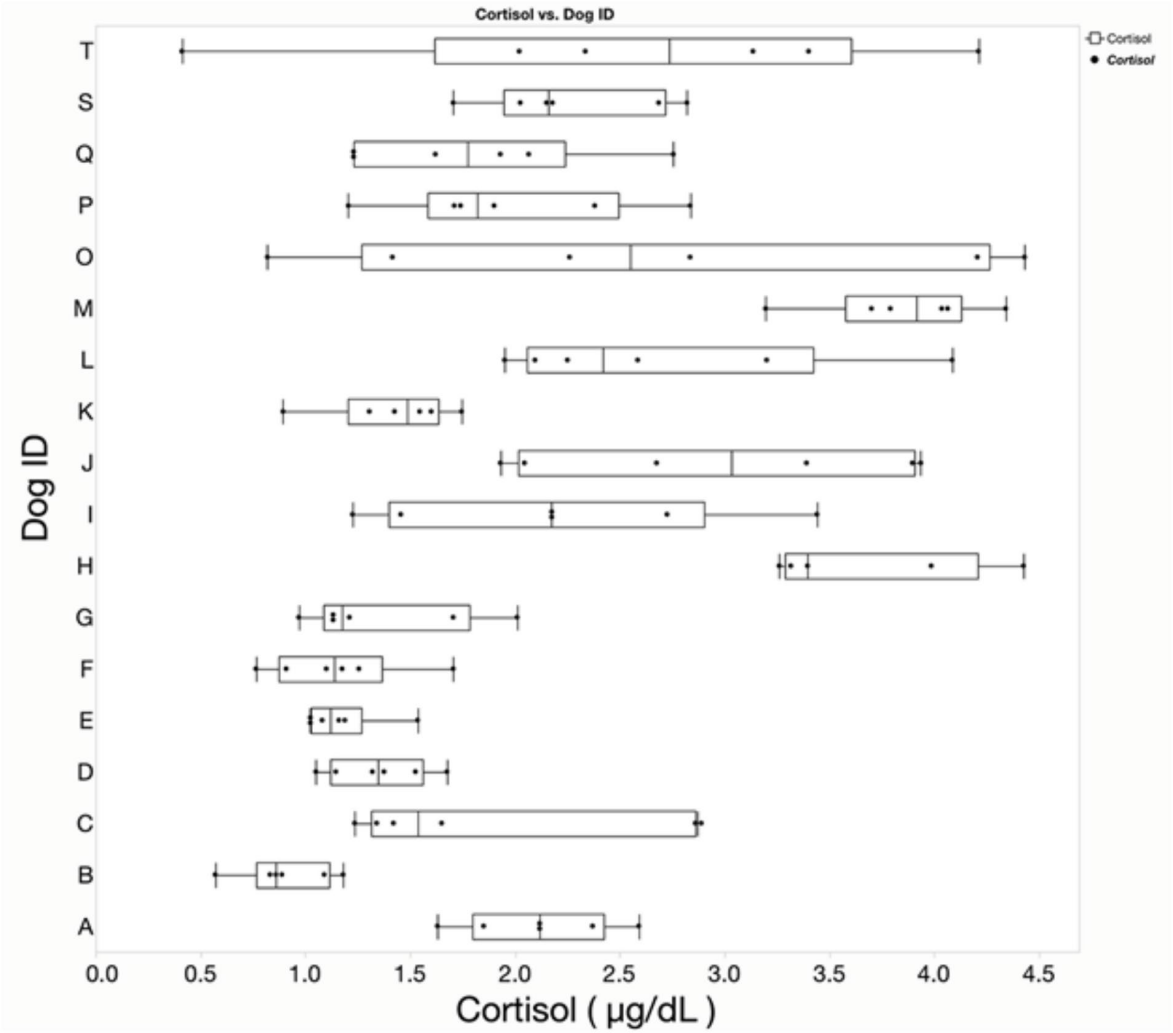
Box-and-whisker plot displaying cortisol concentrations for samples collected from the jugular vein for 6 consecutive weeks in 18 healthy dogs (A–T). The plot illustrates the median, interquartile range (IQR), and minimum/maximum values, illustrating the variability over the 6 weeks study.

Mean cortisol values, calculated as the mean from duplicate measurements for each collection for each dog over the 6-week period, are presented in Table 1. Mean baseline cortisol concentrations did not differ by week across the 6 weeks of the study. Age, sex, weight and reproductive status did not affect mean cortisol concentrations. The baseline cortisol of each dog fell within population reference intervals (pRIs) of our institution for each of the 248 assays (pRI: 1.0–5.9 μg/dL or 27.6–162.8 nmol/L). Figure 1 depicts the results from the 18 dogs, including the minimum and maximum values, lower and upper quartiles, and median for each dog. Mean, SD, Variance, CV_G_, CV_I_, and CV_A_ are reported in Table 2.

**Table 2:** Descriptive statistics of repeated basal cortisol concentrations in healthy dogs over a 6-week period.

	Mean	SD	Variance	CV%		95% CI (lower)	95% CI (upper)
Analytic	2.111	0.145	0.021	CV_A_	6.86	6.03%	7.92%
Group/between	2.112	0.796	0.634	CV_G_	37.70	27.41%	60.32%
Individual/within	2.097	0.680	0.462	CV_I_	32.41	28.18%	38.13%

CV_A_ analytical variability; CV_I_ Individual variability; and CV_G_ Group variability.

**Figure 2:**
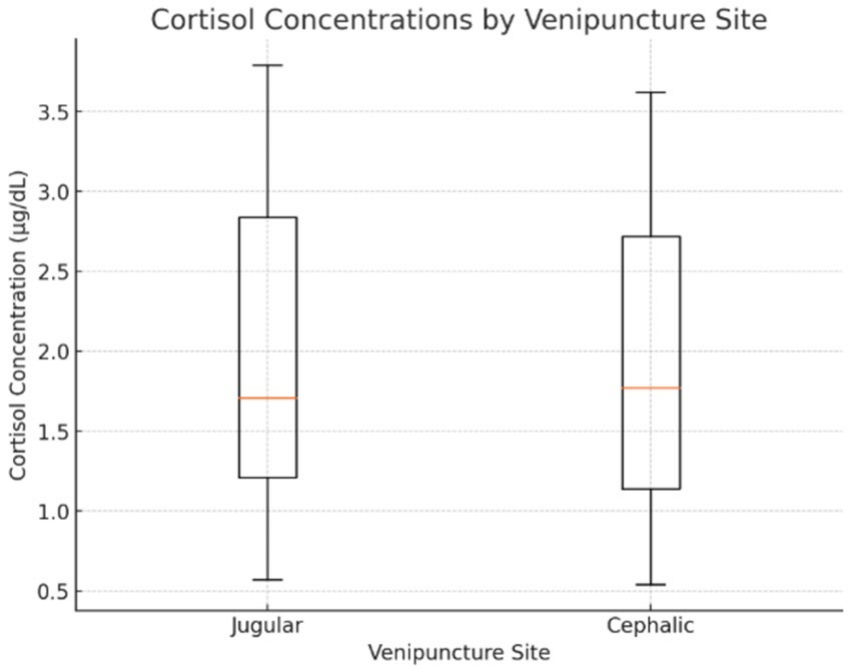
Box-and-whisker plot displaying cortisol concentrations for samples collected from the jugular and cephalic veins in healthy dogs. The plot illustrates the median, interquartile range (IQR), and minimum/maximum values, helping visualize the variability between the two venipuncture sites.

Cortisol concentrations were not different between jugular and cephalic venipuncture sites (*p* = 1.96). The order in which blood was drawn from either site did not influence cortisol levels (*p* = 0.665). Figure 2 presents cortisol concentrations for samples collected from the jugular and cephalic veins. No dogs or samples were excluded from this analysis.

## Discussion

Our study revealed substantial variability in basal cortisol concentrations in healthy dogs over time. This degree of within-individual variability (CV_I_ of 32.41%) and between-individual variability (CV_G_ of 37.30%) was not unexpected, as similar biological variation for cortisol has been described in humans (19, 20). However, the biological variation seen here is higher than the CV_I_ of 5% and CV_G_ of 9% for post-ACTH stimulation test cortisol concentrations in dogs recorded by Gal et al. (5). This finding is interesting but not necessarily surprising as endocrine regulation of cortisol through the hypothalamic–pituitary–adrenal (HPA) axis is complex. A few potential variables include effects of central nervous system neurotransmitter levels on corticotropin-releasing hormone (CRH) secretion from the hypothalamus, CRH-mediated ACTH release by pituitary corticotropes, possible co-regulatory effects of other hormones, and the feedback inhibition of high cortisol concentrations on CRH and ACTH release. Exogenously administered ACTH directly stimulates the release of cortisol from the adrenal glands and may diminish some of the variability present during normal physiological control. The degree of variability in an individual’s response to exogenously administered ACTH may be less pronounced than the variability of baseline cortisol concentrations with normal homeostatic mechanisms.

Based on the daily fluctuations in baseline cortisol recorded by Giannetto et al. (13), we consistently collected blood samples within the same few hours in the afternoon when cortisol levels were expected to be at their highest. The intent was to minimize the variability that may be seen with circadian secretion patterns. In humans, daily fluctuations complicate the interpretation of a single basal measurement unless the blood sample is collected at a certain time during the day (21, 22). The time of sampling and potential disruption of normal circadian rhythmic cortisol secretion is an important consideration when testing for hyperadrenocorticism in people (23). If sample collection was randomized throughout the day, the observed CV_I_ and CV_G_ may have been even larger.

It is well established that stress can cause transient spikes in basal cortisol levels, sometimes exceeding RIs (24–27). To minimize the impact of stress on our cohort, we implemented an acclimation visit prior to the start of sample collection as well as an additional acclimation period on each day of collection. This approach aimed to reduce stress-related variability and ensure more reliable cortisol measurements. Over the 6-week study period, baseline cortisol concentrations consistently remained below our institution’s population-based reference interval (pRI) upper limit (pRI = 1.0–5.0 μg/dL) with a maximum recorded level of 4.66 μg/dL (128.62 nmol/L). Additionally, no within or between subject changes in cortisol levels were identified from week 1 to week 6. This aligns with the results of Squair et al. (28), who demonstrated that low-stress, positive handling can help reduce or normalize cortisol levels over repeated exposures, and that appropriate handling techniques can reduce stress-related cortisol elevation. Other factors such as pre-appointment car ride length, time of arrival, temperament, and phlebotomist may have contributed to the observed CV_I_, and it is likely that these factors play a role in baseline cortisol variability in clinical practice.

Cortisol concentrations in our study did not differ between jugular and cephalic venipuncture sites, nor did the order of blood collection influence cortisol levels. This finding is clinically relevant as it suggests that both venipuncture sites can be used without introducing additional pre-analytical variability in baseline cortisol concentrations. Veterinarians might choose the most practical or least stressful site for sample collection based on the individual animal. Still, as studies in both humans and dogs have demonstrated that factors such as body position (e.g., transitioning from sitting to supine) and tourniquet application can significantly impact plasma concentrations of various analytes (29–33), consistent sampling from the same site in a patient is recommended as a best practice for serial monitoring. It is worth mentioning that using a butterfly catheter to obtain samples from the saphenous vein - another common blood-collection method often used to reduce restraint and patient stress - was not evaluated. The potential impact of this site and technique on cortisol concentrations remains unknown.

No differences were found based on age, sex, weight, or reproductive status. This contrasts with data reported by Gal et al. (5), who found significantly higher mean plasma cortisol concentrations after ACTH administration in males relative to females. This discordance could be related to sampling conditions (after ACTH administration) or differences between sampling populations, as that study sampled within a colony of Harrier Hounds. It could also be that the lesser degree of biological variation in the Gal study (CV_I_ of 5% and CV_G_ of 9%) may have revealed associations that were obscured in our study due to the relatively small and diverse sample set with robust CV_I_ and CV_G_. Future research should continue to explore these possible relationships.

Another limitation of the study was the use of privately owned dogs. Due to private ownership and associated variability in the lifestyle of each dog, day-to-day routine leading up to presentation for blood collection could not be standardized across the group. Instead, the individual routine of each dog, as controlled by their owner, was kept consistent week-to-week as well as on the day of blood draw. For example, if one dog went on a run on the morning of their first blood draw, that dog then needed to go on a run in the morning before each subsequent blood draw. Compliance to routine was assessed with weekly owner interviews. Additionally, due to the voluntary enrollment of privately owned dogs, there was only one intact male and one intact female dog in the study. Although this larger representation of age, weight, breed, and lifestyle is more reflective of a normal clinical population, it may have contributed to the between-individual variability observed. Future studies may benefit from a more balanced cohort of intact to non-intact male and female dogs, and larger cohorts may allow stratification by sex or breed for more targeted reference intervals and biological variation data. The addition of behavioral assessment to further investigate the correlation of cortisol levels and behavior may also provide some insight into the role of stress on large between-subject variation.

Interestingly, 6 of the 18 dogs in our sample set consistently had cortisol concentrations at or below 2 μg/dL. Two additional dogs had concentrations below 2 μg/dL four out of 6 weeks. Several studies have investigated the use of baseline cortisol concentrations in the diagnosis of hypoadrenocorticism in dogs using different analytical methods (1–4). These studies have consistently demonstrated that while dogs with baseline cortisol above 2 μg/dL are unlikely to have hypoadrenocorticism, this cut-off has a poor positive predictive value for hypoadrenocorticism in dogs with cortisol less than 2 μg/dL. Our observations are consistent with these prior studies and reinforce current consensus statements regarding the diagnosis of hypoadrenocorticism in dogs (34). Veterinarians should not use low baseline cortisol alone for the positive diagnosis of hypoadrenocorticism but should instead rely on a combination of clinical signs, other biochemical results, and the ACTH stimulation test.

Biological variation data can be used by laboratories to set analytical quality goals (17). While biological variability unavoidably contributes to clinical uncertainty around a measured value, laboratories may set quality goals to minimize the analytical contribution to this uncertainty. For instance, it has been demonstrated that if CV_A_ ≤ 0.5 × CV_I_ it contributes to less than 12% of the total variability—a standard known as “desirable imprecision” (35–37). More stringent goals with lower ratios of CV_A_ to CV_I_ may be set as appropriate for the analyte in question. In our study the CV_A_ was < 0.25 × CV_I_ near the clinically significant cut-off of 2 μg/dL, a quality goal sometimes referred to as “optimum” imprecision (17). This reinforces the reliability of analytical measurements and suggests that analytical variability is an insignificant contributor compared to individual variation when measuring baseline cortisol concentrations. It should be noted that CV_A_ may vary greatly between analytical methods and between analyzers using the same method. When interpreting patient data in the context of biological variation, the laboratory’s own CV_A_ at or near the level of results of clinical interest should be used.

A recent study by Manzocchi and van Rooyen explored the use of pRIs to derive what they termed empirical biological variation (2024). The goal of the study was to find a mathematical estimation of biological variation (CV_I_ and CV_G_) from a pRI to guide the establishment of analytical performance goals when biological variation data is unavailable. This empirical biological variation (EBV) derived from the pRI was compared to combined biological variation (CV_B_) calculated using CV_B_ = √(CV_I_^2^ + CV_G_^2^). An estimation of EBV for baseline cortisol using their calculations and our institutions pRI is 48%, which is quite similar to the CV_B_ calculated from our data of 50%. Analytical quality goals for baseline cortisol set using the EBV model would be more stringent than goals established using our BV findings, as outlined in Table 3. While Manzocchi et al. outline many pitfalls and limitations of the EBV approach, these findings highlight its potential utility when other evidence-based analytical performance specifications are lacking.

**Table 3:** A comparison of analytical quality goals for baseline cortisol concentrations in dogs established based on biological variation data obtained from 18 healthy, acclimated dogs over a 6-week period and analytical performance goals established using empirically estimated biological variation derived from the population reference interval.

Biological variation from healthy dogs	Empirical biological variation approach using the pRI
CV_B_ = 49.7%	CV_E_ (from pRIs) = 47.7%
Optimal imprecision (CV_A_) goal = < 8.1% (CV_I_ * 0.25)	pCV_A_ goal < 6.89%
Optimal Bias_A_ goal < 6.21%	pB goal < 4.82%
Optimal TEa goal < 19.58%	pU < 16.46%

Optimal imprecision, optimal bias, and optimal total allowable error (TEa) were derived using equations reported by Flatland et al. (17). Permissible imprecision (pCV_A_), permissible bias (pB), and permissible expanded, combined measurement uncertainty (pU) were calculated using equations reported by Manzocchi and Van Rooyen (38).

## Conclusion

Between-individual and within-individual biological variability contributes significantly to measured serum basal cortisol concentrations in dogs and should be considered when interpreting patient data. Serum baseline cortisol concentrations were not significantly affected by age, sex, weight, or neuter status, but associations could have been obscured by biological variability in our small and diverse sample set. Future studies should continue to explore these possible associations. Venipuncture site had no effect, and veterinarians should prioritize patient comfort over concerns for preanalytical variability when choosing a venipuncture site. Baseline cortisol concentrations do not decrease over sequential office visits when dogs are acclimated. Veterinarians should be aware of the effect of biological variation on basal cortisol measurements in dogs when interpreting patient data.

## Data Availability

The original contributions presented in the study are included in the article/supplementary material, further inquiries can be directed to the corresponding author.
